# Home Language Will Not Take Care of Itself: Vocabulary Knowledge in Trilingual Children in the United Kingdom

**DOI:** 10.3389/fpsyg.2017.01358

**Published:** 2017-08-10

**Authors:** Karolina Mieszkowska, Magdalena Łuniewska, Joanna Kołak, Agnieszka Kacprzak, Zofia Wodniecka, Ewa Haman

**Affiliations:** ^1^Faculty of Psychology, University of Warsaw Warsaw, Poland; ^2^Institute of Psychology, Jagiellonian University Kraków, Poland

**Keywords:** trilingual language acquisition, trilingual children, multilingualism in migrant context, vocabulary acquisition, minority language, home language

## Abstract

Language input is crucial for language acquisition and especially for children’s vocabulary size. Bilingual children receive reduced input in each of their languages, compared to monolinguals, and are reported to have smaller vocabularies, at least in one of their languages. Vocabulary acquisition in trilingual children has been largely understudied; only a few case studies have been published so far. Moreover, trilingual language acquisition in children has been rarely contrasted with language outcomes of bilingual and monolingual peers. We present a comparison of trilingual, bilingual, and monolingual children (total of 56 participants, aged 4;5–6;7, matched one-to-one for age, gender, and non-verbal IQ) in regard to their receptive and expressive vocabulary (measured by standardized tests), and relative frequency of input in each language (measured by parental report). The monolingual children were speakers of Polish or English, while the bilinguals and trilinguals were migrant children living in the United Kingdom, speaking English as a majority language and Polish as a home language. The trilinguals had another (third) language at home. For the majority language, English, no differences were found across the three groups, either in the receptive or productive vocabulary. The groups differed, however, in their performance in Polish, the home language. The trilinguals had lower receptive vocabulary than the monolinguals, and lower productive vocabulary compared to the monolinguals. The trilinguals showed similar lexical knowledge to the bilinguals. The bilinguals demonstrated lower scores than the monolinguals, but only in productive vocabulary. The data on reported language input show that input in English in bilingual and trilingual groups is similar, but the bilinguals outscore the trilinguals in relative frequency of Polish input. Overall, the results suggest that in the majority language, multilingual children may develop lexical skills similar to those of their monolingual peers. However, their minority language is weaker: the trilinguals scored lower than the Polish monolinguals on both receptive and expressive vocabulary tests, and the bilinguals showed reduced expressive knowledge but leveled out with the Polish monolinguals on receptive vocabulary. The results should encourage parents of migrant children to support home language(s), if the languages are to be retained in a longer perspective.

## Introduction

The issue of how language input affects language acquisition in monolingual children has been a focus of broad scientific interest (e.g., Hart and Risley, 1995; Rowe, 2012; see Hoff, 2006 for review). Similarly, many studies have looked at how bilingual upbringing impacts the patterns of language input and how bilingual input influences language acquisition, especially in the area of vocabulary development (e.g., De Houwer, 2007; Gathercole and Thomas, 2009; Thordardottir, 2011; Hoff et al., 2012; Hoff and Core, 2013; Gollan et al., 2015; Unsworth, 2016).

The emerging field of investigating trilingual children’s vocabulary acquisition has been largely dominated by case studies and has reported few comparisons with bilingual and monolingual performance. In the present paper we focus on trilingual children and explore their receptive and productive vocabulary in the community^1^ language (English) and one of their home languages (Polish), in comparison with their bilingual and monolingual peers. We also investigate the properties of language input in trilingual children compared to bilinguals. We first briefly discuss what is known about the impact of language input on monolingual language acquisition and then present the available evidence on bilingual and trilingual language acquisition. As the issue of language development in trilinguals is still understudied, the rationale for the present analysis draws considerably on the evidence gathered from research on bilingual child development.

Research on monolingual language acquisition shows that quantity and quality of language input^2^ in child’s environment influence the pace of language development. In a ground-breaking study, Hart and Risley (1995) identified a group of monolingual children with diminished language input (caused indirectly by low family income and low parental education), who, at the age of 3, were estimated to hear 30 million fewer words than their peers from upscale families and had a significantly smaller vocabulary size. A follow-up study on the same children at the age of 9 revealed that the two groups grew further apart in their vocabulary knowledge and, accordingly, in their school performance, as measured by tests of listening, speaking, semantics, and syntax (Hart and Risley, 2003). The results of the studies by Hart and Risley show clearly that the amount of language input a child receives bears consequences for their language attainment and later school outcomes. Since then, researchers have further investigated the role of input in child language acquisition. Rowe (2012) discovered that the quantity of parental input alone was insufficient in developing child’s vocabulary at a preschool age, and identified that the diversity of parental vocabulary and use of decontextualized language (e.g., narratives) were the best predictors of pre-schoolers’ vocabulary growth. Essentially, both quantity and quality of language input have been shown to influence the pace of child’s language acquisition, including vocabulary development (Goodman et al., 2008; Unsworth, 2012, 2013).

Natural variation and diversity present in the language input of bilingual children may impact their vocabulary acquisition. In bilingual children, the quantity of input they receive is naturally divided between two languages, e.g., mother’s vs. father’s language, or L1 (i.e., home, heritage, or minority language) vs. L2 (i.e., community, or majority language). Thus, the nature of bilingual upbringing results in less input for each of the languages in comparison to the input received by monolingual peers (Pearson et al., 1993; Montrul, 2008, but cf. De Houwer, 2014). Reduced language input may be one of the reasons why bilingual children are repeatedly shown to score lower than monolinguals on vocabulary tasks in the majority language (e.g., Leseman, 2000; Oller et al., 2007; Bialystok et al., 2010; Bohnacker et al., 2016). Importantly, those vocabulary setbacks are found in different language pairs (e.g., Bialystok et al., 2010; Klassert et al., 2014; Bohnacker et al., 2016), across pre-school and school years (Bialystok et al., 2010), and – in the case of the majority language – are largely related to home-context vocabulary, rather than the school-context (Bialystok et al., 2010). A direct link between bilingual vocabulary development and language exposure was investigated by Thordardottir (2011) in a group of 5-year-old simultaneous French–English bilinguals in Canada^3^. Bilinguals’ performance on receptive and expressive vocabulary was compared to that of their monolingual peers matched on age, socioeconomic status, and non-verbal intelligence, but differing in the amount of exposure they received in each language. A robust relationship was found between the amount of exposure to a language and children’s performance in that language, although the relationship was observed to be different for the receptive and expressive vocabulary. Bilinguals exposed to both languages to the same extent scored comparably to monolingual children in the receptive vocabulary test, but they needed more input in a given language (and relatively less in the other one) to keep up with their monolingual peers in expressive vocabulary.

Access to many speakers of a given language seems to be another important factor contributing to language abilities in bilinguals. In a recent study by Gollan et al. (2015), the number of heritage language speakers that participants spoke to, correlated positively with their scores on a picture naming task (measured as the number of correct responses) in that language, and did not correlate negatively with their correctness in picture naming in English (community language). Importantly, the effect was independent of how frequently the participant used each language. Presumably, the greater the number of unique native speakers that a child interacts with on daily basis, the greater the variety of words used with a child, which may contribute to the child’s vocabulary.

As demonstrated by the examples above, bilingual language development is a complex and dynamic process, influenced by, among others, the amount of input received in each language, and the number of native speakers of each language that the child has contact with. However, those factors vary in time and can change throughout the course of the child’s development, resulting in shifts in language dominance. For instance, when bilingual upbringing is set in a migration context, the home language is usually the dominant one during the first years of the child’s life. But when the child enters pre-school or school and the exposure to the community language increases, language dominance tends to shift toward the community language. In a questionnaire study aimed at determining factors that influence home language maintenance, De Houwer (2007) analyzed parental language use patterns from almost 2000 bilingual families, where at least one of the parents spoke a heritage language (different than the majority one). She asked how many of the children spoke the heritage language and found that nearly 25% of the children did not. De Houwer traced the origin to the parental language use patterns, showing most families spoke a mix of the heritage and community languages at home. Conversely, a model with the highest chances of successful home language maintenance was when at least one parent spoke only the heritage language at home. This is in line with the 20% threshold hypothesis (Pearson et al., 1997), which suggests that children who hear less than 20% of their input in a given language, are often reluctant to speak that language. According to Hoff et al. (2012), the 20% is an absolute minimum of input for a child to be able (and willing) to use a language. As established by Thordardottir (2011) in a previously mentioned study with 5 year olds in Canada, bilingual children achieved similar level of expressive vocabulary to that of their monolingual peers in either French or English, if they received 60% of their input in that language (French or English). Similar results are reported by Cattani et al. (2014) for children under the age of three exposed to English as a majority language.

Research on language development in trilingual children is an emerging field and features mostly case studies. Kimbrough Oller (2010), who analyzed all-day recordings from a toddler trilingual with German, English and Spanish, showed that directedness of input in the three languages was strongly predictive of the number of words that the child used in each language. Consequently, the child produced more words in the language that was spoken to her directly, compared to the language heard by the child, but not addressed to her. Hoffmann (1985, 2001) spent 7 years observing two early trilingual children, both of whom acquired Spanish and German from their parents, and English, their third language, from the community, school, and peers. The study showed that the children developed “sufficient competence in all three languages to fulfill their communication needs as they were at the time” (Hoffmann, 2001, p. 3). Montanari (2009) found that a Tagalog–Spanish–English trilingual child was able to select the appropriate language according to the interlocutors’ linguistic repertoire before the age of two and that the occasional instances of inappropriate language use were mostly due to vocabulary gaps. Observing the same child (Montanari, 2010), she found that the child’s cumulative vocabulary growth from 1;4 to 2;0 was fairly comparable to that of bilinguals’ and monolinguals’ tested by Pearson et al. (1993). The conclusions from the case-studies of trilinguals are in-line with research on bilingual development, but there is still a need for more extended investigations on larger samples.

Our goal was to examine the vocabulary knowledge in migrant children (lower primary school) who have frequent contact with three languages, and to map the outcomes of vocabulary tests onto the patterns of language use reported by children’s parents. The specific aims were the following:

(1) To investigate the vocabulary knowledge in migrant trilingual children and compare it with the knowledge of their bilingual and monolingual peers.
(2) To explore the relative frequency of input in each language in trilingual families and contrast these patterns with those in bilingual families.
(3) To link language input to vocabulary knowledge in multilingual children.

We explored the actual performance on the receptive and expressive vocabulary tests in trilingual children, and compared those with the lexical performance of bilingual and monolingual peers. We then viewed those results in the light of relative frequency of input in each language in trilingual children and compared it with the input reported in bilingual peers.

## Materials and Methods

### Participants

We analyzed data gathered in a larger project on cognitive and language development of Polish bilingual children (related to COST Action IS0804 ^4^). The database collected in the Bi-SLI-PL project consists of data from 173 bilingual children living in the United Kingdom who had at least one Polish parent, 311 Polish monolingual children, and 30 English monolingual children. A written parental consent was obtained for all the children participating in the study. In addition to the vocabulary testing, participants completed a large battery of tools measuring grammar knowledge, phonological processing and storytelling, however, the results of these tests are beyond the scope of this paper.

For the current analyses, we used data from 56 children, trilingual, bilingual, and monolingual. We first selected all children who had been exposed to more than two languages (*n* = 14). These children, i.e., the trilingual group living in the United Kingdom, were born to families with one Polish parent and one parent of other nationality, so they were exposed to two home languages from birth: Polish and another language (Albanian/Arabic/Bengali/French/Italian/Macedonian/Russian/Ukrainian), and to the majority language, English (age of onset: *M* = 8 months, *SD* = 14 months, range: 0–36 months). The selected group of participants was matched (in a one-to-one pairwise fashion) with the peer groups of: (1) Polish–English bilinguals living in the United Kingdom (*n* = 14); (2) Polish monolinguals living in Poland (*n* = 14); (3) English monolinguals living in the United Kingdom (*n* = 14). The pairwise matching was based on the chronological age, gender, and the non-verbal intelligence score. We also compared the children’s socio-economic status (measured in the years of maternal education). A Kruskal–Wallis test showed no statistically significant difference in SES between the four groups, H(3) = 5.7, *p* = 0.125 (see **Table 1** for details).

**Table 1:** Characteristics of the participants: gender, age (in months), non-verbal intelligence score and maternal education (in years) across trilinguals, bilinguals, Polish monolinguals, and English monolinguals.

	Trilinguals (*n* = 14)	PL-EN bilinguals (*n* = 14)	PL monolinguals (*n* = 14)	EN monolinguals (*n* = 14)	Between-groups comparison
Gender	6 m + 8 f	6 m + 8 f	6 m + 8 f	5 m + 9 f	χ^2^(3) = 0.22, *p* = 0.975
Age (months) M ± SD	66 ± 9	66 ± 9	67 ± 9	66 ± 9	H(3) = 0.03, *p* = 0.999
Raven (raw score) M ± SD	23 ± 5	23 ± 4	23 ± 5	21 ± 4	H(3) = 2.18, *p* = 0.537
Maternal education (years of schooling) M ± SD	15 ± 3	17 ± 3	16 ± 3	17 ± 2	H(3) = 5.7, *p* = 0.125

### Procedure

The data analyzed here were gathered in the Bi-SLI-PL project (related to COST Action IS0804). The project used a number of measures of linguistic and cognitive development (see Haman et al., Unpublished). The present analysis focuses on vocabulary measures (receptive and expressive vocabulary size) and the parental reports of the child’s input in each of their languages.

#### Expressive and Receptive Vocabulary Tests

We used standardized picture-naming and word-recognition tests in Polish and English in the case of bilinguals and trilinguals, or in one of those languages in the case of monolinguals. For English, we applied the Expressive Vocabulary Test (Williams, 2007) to assess the children’s expressive word knowledge, and for Polish we used Zadanie Nazywania Obrazków (Haman and Smoczyńska, 2010, Unpublished). In both tests of expressive vocabulary we asked the children to name pictures illustrating objects (for nouns as target words), their features (adjectives), or some activities (verbs). Receptive word knowledge was assessed with the British Picture Vocabulary Scale (BPVS-3; Dunn et al., 2009) in English, and Obrazkowy Test Słownikowy – Rozumienie (OTSR; Haman et al., 2012) in Polish. In both tests of receptive vocabulary children were asked to choose one picture depicting the target word out of four colorful pictures presented on each board. The raw scores from the tests were transformed into standard scores (z-scores). The mean score and the standard deviations were calculated on the monolingual populations (monolingual Polish for the Polish vocabulary tests, and monolingual English for the English vocabulary tests). Using standard scores allowed us to establish how far from the monolingual mean were the scores of the bilingual and trilingual groups.

#### Parental Reports of Input in Each of the Child’s Languages

We used a Polish version of the Questionnaire for Parents of Bilingual Children^5^ [(PABIQ – Tuller, 2015; Polish adaptation by (Kuś et al. 2012, Unpublished)] to extract the information about the number of speakers and the frequency of bilingual and trilingual children’s input in the home and majority languages^6^. Specifically, we asked parents to estimate on a five point Likert scale how often (and with whom) their child was addressed in each language in specific communicative situations in two types of settings: at home and outside of home.

The communicative situations at home (henceforth referred to as at-home input) included two factors with different weights: we asked the parents to estimate how often each language was used toward the child by each of the parents and the siblings [from 0 = “never,” 2 = “rarely,” 4 = “sometimes,” 6 = “most of the time,” 8 = “always,” maximum score: three sources (mother, father, siblings) ^∗^ 8 “always” = 24 points]. We also asked them to specify how often each language was used toward the child by the grandparents and the possible care-takers (e.g., babysitter), and was used in the parent-to-parent interaction (i.e., language not directed toward the child but which can still be overheard by the child) [from 0 = “never,” 1 = “rarely,” 2 = “sometimes,” 3 = “most of the time,” 4 = “always,” maximum score: four sources (grandparents, babysitter, mother speaking to father, father speaking to mother) ^∗^ 4 “always” = 16 points]. Thus, the input from the parents and siblings was weighted more than the input from other adults close to the family. The maximum total score on the index of at-home input was 40 points (24 + 16) for each language. The higher the number of the speakers in a particular language, the higher the total score of input in that language (accordingly, the total score was proportionately lower if child did not have contact with their grandparents and/or did not have siblings). To allow an approximate assessment of the relative contribution of each language into a child’s language input, the total score in each language was transformed into a percentage value. For instance, to get a percentage value for Polish, we divided the total score for Polish by the sum of the total scores for all child’s languages, and multiplied it by 100^7^.

The communicative situations outside of home (henceforth referred to as outside-of-home input) included a number of factors with different weights: the number of hours spent at school divided by 3 (maximum score: 36 h/3 = 12 points), participation in after-school activities in each language (from 0 = “never,” 1 = “once a week,” 2 = “everyday,” maximum score: 2 points), frequency of book reading, storytelling, rhymes/singing, computer games, TV/movies watching in each language (from 0 = “never,” 1 = “once a week,” 2 = “everyday,” maximum score: five activities ^∗^ 2 “everyday” = 10 points). Parents also estimated how often each language was used toward the child by their peers (from 0 = “never,” 2 = “rarely,” 4 = “sometimes,” 6 = “most of the time,” 8 = “always,” maximum score: 8 points), and by family guests and/or relatives not living in the house [from 0 = “never,” 1 = “rarely,” 2 = “sometimes,” 3 = “most of the time,” 4 = “always,” maximum score: two sources (family guests, relatives) ^∗^ 4 “always” = 8 points]. The maximum total score for the frequency of outside-of-home input was 40 points for each language. The higher the number of activities/additional speakers in a particular language, the higher the total score of input in that language (accordingly, the total score was proportionately lower if child did not attend any extracurricular/listed activities, or the family did not have any regular visitors). The total scores in each language were transformed into percentage values (i.e., to get a percentage value for Polish, we divided the total score for Polish by the sum of the total scores for all child’s languages, and multiplied it by 100).

#### Testing Procedure

The children were tested by a native or near-native speaker of the language (Polish or English) in a quiet room: the monolingual children in their preschools, the bilingual and trilingual children in their day-cares, schools or in their homes in the United Kingdom. The bilingual and trilingual children were tested by different experimenters, and on different days in each of their respective languages. They were first tested in their dominant language (either Polish or English, as reported by the parents), and then in the other language. There was a maximum of a 6-week break between the two language testing sessions.

### Statistical Analysis

Given the small size of each sample (*n* = 14), we employed non-parametric tests of group differences to tackle potential violation of normality assumption in ANOVA. We performed a series of Wilcoxon–Pratt Signed-Rank Test to compare amount of contact with home and majority languages between bilingual and trilingual children. We used Kruskal–Wallis tests to contrast the receptive and expressive vocabulary knowledge of trilingual, bilingual, and monolingual children. Whenever the Kruskal–Wallis tests revealed significant differences between the groups, we used Nemenyi test as *post hoc.*

## Results

### Vocabulary of Trilinguals

The main aim of the current analysis was to examine the vocabulary knowledge in trilingual children in comparison with the bilingual and monolingual groups.

#### Vocabulary in English

**FIGURE 1:**
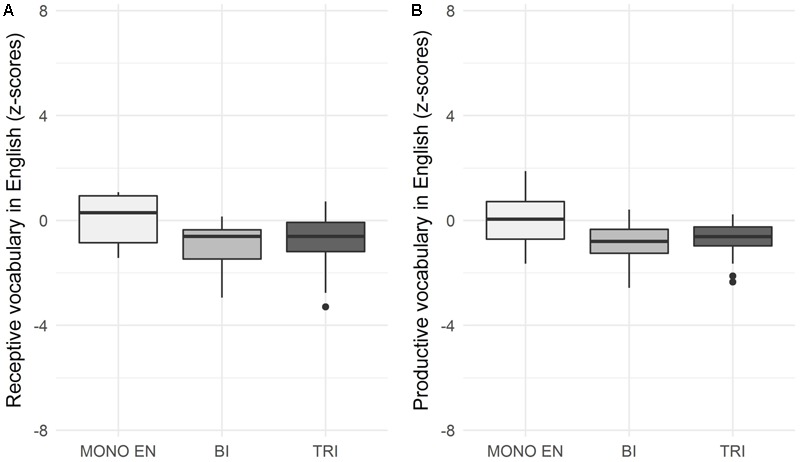
Receptive **(A)** and productive **(B)** vocabulary in English (z-scores) across three groups: monolingual English, bilingual, and trilingual children.

The trilinguals’ raw scores on receptive and productive vocabulary tests in English were compared to those of the bilinguals and English monolinguals. The descriptive results are presented in **Figure 1**.

We used the Kruskal–Wallis test to compare the English receptive vocabulary scores across the three groups. We found no significant effect of group, H(2) = 4.81, *p* = 0.09.

The Kruskal–Wallis test used to compare English productive vocabulary scores across the three groups showed a marginally significant effect of group, H(2) = 6, *p* = 0.049. However, a Nemenyi *post hoc* revealed no significant difference between the groups (trilinguals vs. bilinguals: *p* = 0.956, trilinguals vs. English monolinguals: *p* = 0.119, bilinguals vs. English monolinguals: *p* = 0.062).

#### Vocabulary in Polish

**FIGURE 2:**
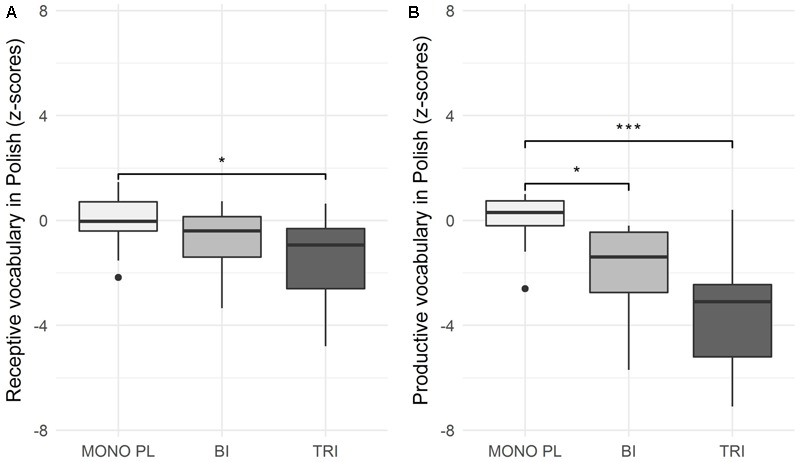
Receptive **(A)** and productive **(B)** vocabulary in Polish (z-scores) across three groups: monolingual Polish, bilingual, and trilingual children. ^∗^*p* < 0.05, ^∗∗^*p* < 0.01, and ^∗∗∗^*p* < 0.001.

The trilinguals’ raw scores on the receptive and productive vocabulary tests in Polish were compared to those of the bilinguals and Polish monolinguals. The descriptive results are presented in **Figure 2**.

The Kruskal–Wallis test was used to compare the Polish receptive vocabulary scores across the three groups, and showed a significant effect of group, H(2) = 8.23, *p* = 0.016. A *post hoc* analysis revealed that the Polish monolinguals scored significantly higher on the receptive test in comparison with the trilinguals (*p* = 0.012). We found no significant differences between the receptive Polish vocabulary scores of the Polish monolinguals and bilinguals (*p* = 0.279). Furthermore, we found no statistically significant difference between the scores of the bilinguals and trilinguals (*p* = 0.373).

Again, we used the Kruskal–Wallis test to compare the Polish productive vocabulary scores across the three groups. We found a significant effect of group, H(2) = 21.89, *p* = 0.001. A *post hoc* analysis revealed that Polish monolinguals scored significantly higher on productive vocabulary in comparison with trilinguals (*p* = 0.001), and bilinguals (*p* = 0.013). Though the trilinguals’ average score was numerically lower than that of the bilinguals and Polish monolinguals, we found no statistically significant difference between the scores of the trilinguals and bilinguals (*p* = 0.166) on the productive vocabulary size in Polish.

### Language Use Patterns in Bilingual and Trilingual Families

**FIGURE 3:**
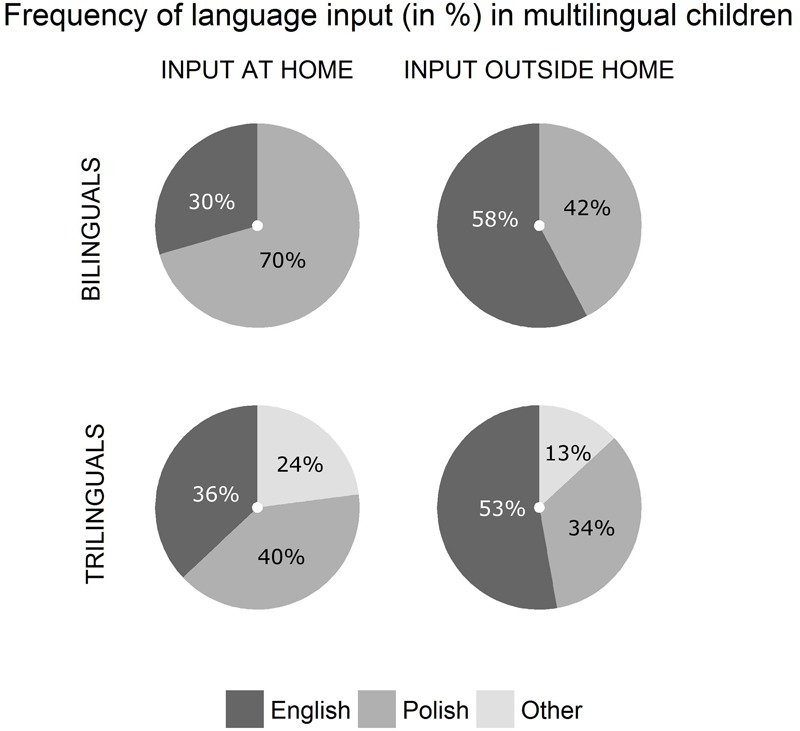
Relative frequency of at-home input (in %) and outside-of-home input in Polish, English, and Other language across the bilingual and trilingual groups.

In order to examine the vocabulary results in view of the language environment of our participants, we compared the frequencies of input in each language in the bilingual and trilingual groups. We focused our comparison on those two groups because we were interested specifically in the language use patterns in the bilingual and trilingual families. In the case of Polish and English monolingual children, we assumed their input was wholly in their native language. The descriptive results from the bilingual and trilingual groups are given in **Table 2** and presented in **Figure 3**.

**Table 2:** The frequency of at-home input and outside-of-home input (in %) in each language across bilingual and trilingual groups.

	Bilinguals		Trilinguals		
	English (*M ± SD*)	Polish (*M ± SD*)	English (*M ± SD*)	Polish (*M ± SD*)	Other (*M ± SD*)
At-home input (%)	30 ± 19 Range: 0–59	70 ± 19 Range: 41–100	36 ± 18 Range: 0–61	40 ± 13 Range: 16–63	24 ± 11 Range: 11–48
Outside-of-home input (%)	58 ± 8 Range: 43–74	42 ± 8 Range: 26–57	53 ± 7 Range: 41–71	34 ± 7 Range: 17–44	13 ± 6 Range: 4–21

#### At-home Input

While the bilinguals’ at-home input was predominantly Polish, the frequency of the trilinguals’ at-home input was more equally distributed between the three languages: Polish, English and Other (see **Table 2** and **Figure 1**). Wilcoxon–Pratt Signed-Rank Test showed that the trilinguals and bilinguals differed significantly in the relative frequency of input in Polish (*W* = 15, *Z* = -2.75, *p* = 0.003), with the trilinguals hearing Polish less frequently, relative to bilinguals. However, there was no difference between the groups on the frequency of input received in English (*W* = 102.5, *Z* = 0.7, *p* = 0.519).

#### Outside-of-Home Input

The two groups heard English spoken in the outside-of-home context equally frequently (*W* = 49, *Z* = -1.49, *p* = 0.151). Also, outside of home, the two groups heard English more frequently than any other language. However, the Wilcoxon–Pratt Signed-Rank Test showed that the two groups differed significantly in the frequency of the outside-of-home input in Polish (*W* = 38, *Z* = -2.43, *p* = 0.012), with the trilinguals hearing Polish less frequently than the bilinguals.

Overall, the results on language use patterns in bi- and trilingual homes reveal that while the two groups heard English equally frequently, they differed significantly in the frequency of the input in Polish, with the trilinguals having less frequent contact with Polish than the bilinguals.

### Linking Vocabulary Scores and Frequency of Input

**FIGURE 4:**
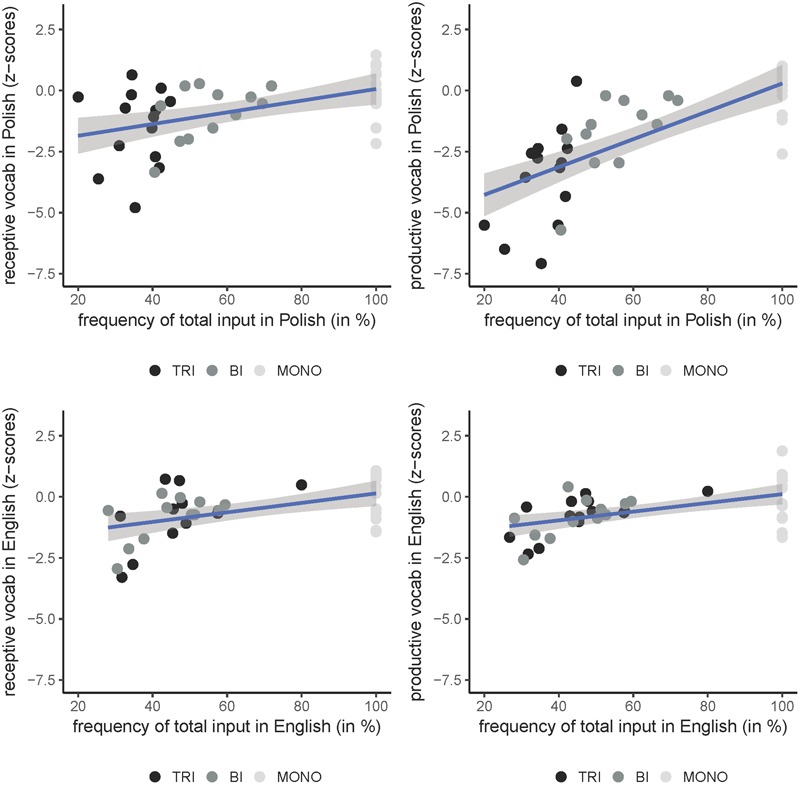
The distribution of vocabulary scores (receptive and productive) of individual children from the three groups in relation to the relative frequency of total input (at home and outside of home) received in English and Polish. The lines represent lines of best fit with the 95% confidence level interval.

Finally, we investigated the relationship between the vocabulary scores and the relative frequency of the input received in English and Polish. For this purpose, we used a combined index of language input which was a sum of the input at-home and outside-of-home. A series of Spearman’s rank correlations assessed the relationship between the vocabulary scores in English and Polish and the relative frequency of the input received in the two languages. The correlations were done on data from all the subjects, with no differentiation between the trilingual, bilingual, and monolingual groups. **Figure 4** presents the correlations, separately for English and Polish and for the receptive and productive vocabulary scores. In English, the correlation between the relative frequency of total input received in English and the vocabulary scores and was *r_s_* = 0.539, *p* < 0.001 for the productive vocabulary, and *r_s_* = 0.451, *p* < 0.01 for the receptive vocabulary. For Polish, the correlation between the relative frequency of the input in Polish and the vocabulary score was *r_s_* = 0.821, *p* < 0.001 in the productive test, and *r_s_* = 0.503, *p* < 0.001 in the receptive test.

Overall, the results show that the relative frequency of the input received in each language was positively and strongly correlated with the vocabulary scores in this language. In both languages, the correlations were stronger in the domain of the productive vocabulary.

## Discussion

The goal of the paper was to compare lexical knowledge of trilinguals with their bilingual and monolingual peers and to relate the vocabulary outcomes to the daily patterns of language use reported by the parents. To this aim, we compared the results in their expressive and receptive vocabulary tests with those of carefully matched bilingual and monolingual children. We also analyzed their language outcomes in the light of the relative frequency of input in each of the trilingual children’s languages, and compared them with the input received by their bilingual counterparts.

First, we examined the vocabulary knowledge of trilinguals, Polish–English bilinguals, and Polish and English monolinguals. We compared their receptive and expressive vocabulary scores for Polish (the home language) and English (the community language). For English, the results revealed no significant differences between the bilinguals’ and trilinguals’ vocabulary size on either the receptive, or productive vocabulary tests. Moreover, the two groups did not differ from the English monolinguals in their receptive and productive vocabularies. For Polish, however, the results paint a much more complex picture. The trilinguals and bilinguals showed similar vocabulary scores in both receptive and productive tests in Polish. When we compared the vocabulary knowledge of the two groups with that of Polish monolinguals, differences occurred. With respect to the receptive vocabulary in Polish, we found that while the bilinguals did not differ from the Polish monolinguals, the trilinguals had significantly smaller receptive lexicons. In terms of the productive vocabulary in Polish, both the bilinguals and trilinguals scored significantly lower than the Polish monolinguals. Additionally, we investigated the relationship between the children’s vocabulary scores and the relative frequency of the input received in English and Polish. We found positive strong correlations between the relative frequency of the input received in each language and the vocabulary scores in that language. In both languages, the correlations were stronger in the domain of the productive vocabulary.

Overall, we found two main characteristics of trilingual development in the immigrant context. First, we established that in terms of the majority language (English), the trilingual and bilingual children in our sample showed vocabulary knowledge similar to that of their monolingual peers. This was demonstrated by the lack of significant differences between the three groups in both receptive and productive English vocabulary tests. The current data provide evidence in support of the claim expressed by Grosjean that in a migrant context, the majority language is likely to take care of itself, mostly due to the large exposure to the language in the daycare or school and from the peers (Grosjean, 2010, p. 209). This we have found to be equally true for both the bilingual and trilingual children in our sample.

Secondly, we found that the bilinguals and trilinguals showed significantly lower vocabulary knowledge in their home language Polish, as compared to non-migrant Polish monolingual peers matched on age, gender, and non-verbal IQ. A possible explanation of this finding may lie in the patterns of language use in the multilingual homes. Since the quantity of language input in the child’s environment has been long established as crucial for the pace of language development (Goodman et al., 2008; Rowe, 2012; Hoff, 2013; Gollan et al., 2015), receiving less input in the home language may cause setbacks in developing comprehension and production in that language. To explore the potential impact of language input on the vocabulary knowledge in our sample, we examined the relative frequency of the input in each language in the bilingual and trilingual groups. To this end, the parents of bilingual and trilingual children were asked to specify how often the child is addressed in each of the languages at home (i.e., among family members) and outside of home (i.e., by peers, at school, during after-school activities). The analysis of the questionnaire data revealed that the trilinguals and bilinguals heard English (the majority language) equally frequently at home. Additionally, both groups heard English most frequently outside of home. However, the bilinguals and trilinguals differed significantly in the reported frequency of input in Polish (the home language). The trilinguals in our sample heard Polish less frequently both at home and outside of home, as compared to the bilinguals. The data we gathered do not reflect the absolute amount of input the children received in each of their languages, rather the relative frequency of input received in each language. Nevertheless, we have found that the relative frequency of the input in the home language (Polish), naturally reduced in the bilingual context, was even further limited in the trilingual home, where communication was divided between the two parental languages. Against this background, we have seen a worse performance of the trilingual and the bilingual groups on the Polish vocabulary tests, relative to their Polish monolingual peers.

The present analysis has practical implications for the parents and caretakers of bilingual and multilingual children. The results indicate that the majority language may develop equally well to that of monolinguals, but it is the home language(s) that require(s) more considerate attention. It seems that when the bilingual and the trilingual children enter preschool or school, the exposure to the community language increases, shifting language dominance toward that language. This may eventually lead to developing language abilities predominantly in the community language at the expense of the home language, especially if the home language enjoys lower social prestige than the majority language (Gathercole and Thomas, 2009). To maintain their home languages children need rich and varied home language input. Since the nature of bilingual and trilingual upbringing results in getting less input for each of the languages in comparison to the input received by monolinguals, it is important to maintain the quality of the child directed input. Other studies have demonstrated that of crucial importance to the child’s developing lexicon is not only the mere quantity of input, but also diversity of vocabulary used (e.g., Rowe, 2012), utterance length (Hoff, 2003), and the number of speakers (Gollan et al., 2015). Some researchers, e.g., De Houwer (2007) suggest multilingual families should have at least one parent speaking only the home language, with no code-mixing.

Thus, we would like to call attention to the quality of input a multilingual child receives and we believe it is important to encourage parents and practitioners to invest in all sorts of child-friendly activities (play groups, reading clubs, etc.) as to provide linguistically rich and varied input of the home language(s) in out-of-home context and more opportunities to use the language(s).

It is crucial to stress that the amount and quality of language input a bilingual or trilingual child receives bears an impact on their attainment of the home language. This might turn out particularly important in view of return migration to the parents’ home country, where children often experience educational difficulties, mostly due to the fact that their home language is relatively weaker than their former majority language (Grzymała-Moszczyńska et al., 2015).

### Study Limitations

The presented analysis is not without limitations. The first one is a small size of the compared groups (each group consisted of only 14 children). However, it needs to be stressed that the compared groups were carefully selected and matched. First, to ensure the groups’ homogeneity on potential confounding distracting factors, we employed pair-wise matching to the trilingual group on multiple variables (chronological age, gender, and non-verbal intelligence score). Secondly, the groups did not differ in either age, gender, non-verbal IQ score, or socio-economic status (measured in years of maternal education). Moreover, since our data did not follow normal distribution, we used non-parametric tests for the analyses, the statistical power of which is weaker, i.e., they are less likely to find statistical differences.

Another constraint of the study is the interpretation of the questionnaire data concerning the frequency of language input: the estimation provided by the parents are by definition non-objective and intuitive (e.g., when filling in the questionnaire, parents choose whether a particular language is used toward the child “sometimes” or “most of the time” based on their own interpretations of the scale). Moreover, our index does not account for the variance in the amount of parent–child contact between mothers and fathers (it is possible that the mothers had relatively more contact with the children than the fathers, e.g., the mother’s frequent use of Polish may not equal the father’s frequent use of another language). Nevertheless, the same indices of language input were repeatedly used before (e.g., Blom and Bosma, 2016; Bohnacker et al., 2016; dos Santos and Ferré, 2016; Fleckstein et al., 2016; Rinker et al., 2017) as valid measures of the relative frequency of input in the child’s languages.

Finally, the participants were tested only at one study point. Therefore we were not able to observe the changes in their access to input in the languages over time and the potential changes in their lexical knowledge. Such an analysis would be most informative of the actual retainment of the children’s bilingualism and trilingualism.

## Conclusion

The present analysis aimed to investigate vocabulary knowledge of trilingual migrant children in relation to the reported patterns of their language use. Crucially, we have shown that the majority language (English) of the migrant children may take care of itself, but this is not the case with the home language (Polish). We have linked these results to the relative frequency of the input in Polish and demonstrated that receiving less input in the home language may hinder vocabulary acquisition in that language.

The novelty of the paper was twofold. First, to the best of our knowledge, no previous research has investigated vocabulary acquisition in trilingual migrant children in a group study – previous papers on this topic were for the most part case studies. Second, we have contrasted trilingual language acquisition with language outcomes of bilingual and monolingual peers. We hope that this study will increase the interest in trilingual language acquisition in children and lay foundations for further investigations of the kind.

## Ethics Statement

This study was carried out in accordance with the recommendations of the Ethics Committee at Faculty of Psychology, University of Warsaw, with written informed consent from all subjects. All subjects gave written informed consent in accordance with the Declaration of Helsinki. The protocol was approved by the Ethics Committee at Faculty of Psychology, University of Warsaw.

## Author Contributions

Conception or design of the work: KM, MŁ, JK, AK, ZW, EH; data collection: KM, JK, AK; data analysis and interpretation: KM, MŁ, JK, AK, ZW, EH; drafting the article: KM, MŁ, JK, AK, ZW, EH; critical revision of the article: KM, MŁ, JK, AK, ZW, EH; final approval of the version to be published: KM, MŁ, JK, AK, ZW, EH.

## Conflict of Interest Statement

The authors declare that the research was conducted in the absence of any commercial or financial relationships that could be construed as a potential conflict of interest.

## Footnotes

1 In the present paper we use the terms “L1,” “home language,” “minority language,” and “heritage language” interchangeably, and contrast those with “L2,” “community language” and “majority language.” In the Introduction, whenever we use a specific term, we follow the terminology chosen by the Authors of studies we report.

2 In the present paper we use the terms “input” and “exposure” interchangeably when referring to the measurable interactions with the child in a given language. However, see Carroll (2017) for a call for a clearer definition of (and dissociation between) the terms.

3 Though the bilingual context explored in the study by Thordardottir (2011) does not refer to bilingual migrants (the focus of this paper), the study investigates a direct link between language exposure to vocabulary skills in bilinguals and has thus been considered as relevant to the topic of the present paper.

4 www.bi-sli.org

5 The same questionnaire was used in Haman et al. (Unpublished), to calculate an index of cumulative exposure to L1 and L2 in bilingual children. However, for the present analysis we calculated a different index related to the frequency of input in each of the multilingual child’s languages, and the number of speakers in the child’s linguistic environment.

6 Interpretation of the questionnaire data need to be treated with caution: the parental estimations of frequency of input, as any self-reported measures, are by nature subjective and intuitive. This point is taken up in more detail in Study Limitations. However, we would like to note here that the same indices of language input, or parallel indices based on the same set of questions, have been used by researchers before in studies on different languages (e.g., Blom and Bosma, 2016; Bohnacker et al., 2016; dos Santos and Ferré, 2016; Fleckstein et al., 2016; Rinker et al., 2017).

7 It is important to note, that the indices described above do not indicate the absolute amount of input received in each of the child’s languages, but rather the relative frequency of input received in each language, which is, among others, influenced by the number of speakers in child’s linguistic environment, and regular activities performed in each language.
